# Urinary Retention After GreenLight Laser Photoselective Vaporization of the Prostate (GLL.PVP) Surgery for Benign Prostatic Hyperplasia (BPH): A 3-6 Month Retrospective Follow-Up Study

**DOI:** 10.7759/cureus.73585

**Published:** 2024-11-13

**Authors:** A B Azharul Islam, David Ellis, Natasha Chari, Katie Mccomb, Maisha Zaman Poushi, Ivo Donkov

**Affiliations:** 1 Urology, West Middlesex University Hospital, London, GBR; 2 Surgery, Dhaka Medical College & Hospital, Dhaka, BGD

**Keywords:** bph, catheter, greenlight laser photoselective vaporization of the prostate (gll.pvp), high-risk factors, urinary retention (ur)

## Abstract

Objective

Our study was designed to evaluate the postoperative urinary retention (UR) and success rate of the GreenLight Laser (Boston Scientific, Marlborough, MA, USA) photoselective vaporization of the prostate (GLL.PVP) procedure for Benign Prostatic Hyperplasia (BPH) patients, both with and without high-risk factors.

Methodology

We conducted a retrospective follow-up study of postoperative patients who underwent GLL.PVP for BPH. We collected data from clinical health records, including notes from the lower urinary tract symptoms (LUTS) clinic, trial without catheter (TWOC) clinic, and emergency department presentations with UR. The analysis examined several parameters, including the patient's age, high-risk factors, prostate volume, and both preoperative and postoperative objective voiding parameters. These voiding parameters included post-void residual (PVR) and maximum flow rate (Qmax). Additionally, the analysis looked into whether the patient had a catheter or experienced urinary retention prior to the surgery.

Results

A total of 50 GreenLight Laser PVP surgeries were performed over a 14-month period from May 2023 to July 2024 at West Middlesex University Hospital in London. Most of the patients were between the ages of 60 and 80. Prior to the surgery, data indicated that 17 patients (34%) were using long-term catheters, whereas 33 patients (66%) were not. Additionally, 25 patients (50%) were identified as having high-risk factors, which included being on anticoagulation therapy, a history of urinary retention, and a prostate volume exceeding 100 cc. Notably, the patient without h/o urinary retention had an average preoperative PVR of 150 mL and an average Qmax of 7 mL/second

In our 3-6 month retrospective postoperative follow-up study, we found that eight cases (16%) developed urinary retention, while 42 cases (84%) did not, with an average PVR volume of 105 mL and an average Qmax of 13.5 mL/second. In patients with preoperative urinary retention, 13 cases (76%) did not develop UR postoperatively whereas four cases (24%) developed.

Conclusion

Our study conclusively found that 42 cases (84%) of patients did not experience urinary retention (UR) in the follow-up after undergoing the GLL PVP operation, irrespective of any history of urinary retention or other high-risk factors. This unequivocally demonstrates the operation's efficacy. Furthermore, our findings revealed that three-quarters of patients with a preoperative history of urinary retention also did not develop UR post-surgery. The GLL.PVP procedure is safe and effective, leading to rapid improvements in voiding parameters.

## Introduction

Benign prostatic hyperplasia (BPH) involves the growth of prostatic stromal cells, leading to an enlarged prostate gland. This enlargement compresses the prostatic urethra, restricting urine flow from the bladder. This interference with urine flow can lead to lower urinary tract symptoms (LUTS) such as frequency, urgency, nocturia, intermittency, decreased stream, and hesitancy [1]. BPH affects 50-60% of men aged 60-70 years, with this prevalence rising to 80-90% among men aged 70-80 years [2].

The primary approach to treating this condition includes the use of alpha-blockers, 5-alpha-reductase inhibitors, and anticholinergics, which can be effective both individually and in combination. Clinical trials have demonstrated that combination therapy can lead to significant improvements in LUTS symptoms, IPPS (International Prostate Symptom Score) scores, and peak urinary flow compared to single medication treatments. Additionally, using these medications together can help reduce the risk of urinary retention and minimize the need for prostate surgery. For some patients, however, if these treatments do not provide adequate relief, surgical intervention may be necessary to effectively address bladder outlet obstructions [3, 4]. Failure to address BPH can lead to serious complications, including urinary retention (UR). This, in turn, can cause bladder dysfunction, reduced urine flow rates, renal insufficiency, and urinary tract infections (UTIs). A randomized trial clearly demonstrated that 2.9% of men with moderate BPH symptoms who chose watchful waiting ultimately developed UR [5, 6, 7, 8].

Historically, transurethral resection of the prostate (TURP) has been considered the gold standard for surgery to treat benign prostatic hyperplasia (BPH). However, minimally invasive laser-based procedures, such as holmium laser enucleation of the prostate (HoLEP) and GreenLight laser (Boston Scientific, Marlborough, MA, USA) photoselective vaporization of the prostate (GLL.PVP), have gained popularity. These alternatives are becoming increasingly favored due to their improved safety profiles and comparable functional outcomes [9].

In high-risk groups, such as individuals with urinary retention, prostates larger than 100 mL, and an increased risk of bleeding, there is significant clinical evidence indicating that GLL.PVP is as effective as TURP in managing the symptoms of BPH [10].

The objective of this study is to evaluate the incidence of post-operative urinary retention in patients undergoing GLL.PVP.

## Materials and methods

Study design and setting

This study employed a retrospective cohort design to evaluate the outcomes of GLL.PVP in patients with BPH that causes lower urinary tract symptoms (LUTS). The data collection centered on the historical records of consecutive patients who underwent the GLL.PVP procedure over a 14-month period, spanning from May 2023 to July 2024. The study was conducted at the Urology Department of West Middlesex University Hospital in London, United Kingdom. During the study period, all relevant data were collected from the hospital’s patient records, radiology images, and clinical notes.

Study population, sample size, and study measures

The study population included all consecutive patients who underwent GLL.PVP within the study period. A total of 50 GLL.PVP surgeries were performed over a span of 14 months. The analysis examined several parameters, including the patient's age, high-risk factors, prostate volume, and both preoperative and postoperative objective voiding parameters. These voiding parameters included post-void residual (PVR) and maximum flow rate (Qmax). Additionally, the analysis looked into whether the patient had a catheter or experienced urinary retention prior to the surgery.

Data collection and statistical analysis

Data was collected retrospectively from the hospital's clinical records and databases, including radiology images to determine the prostate volume and clinical health records: LUTS clinic notes, trial without catheter (TWOC) clinic notes, and emergency department presentations for urinary retention.

Statistical analyses, both descriptive and inferential, were conducted to assess the relationships between patient demographics, clinical factors, and surgical outcomes. Descriptive statistics - the mean, median, and standard deviation were calculated for continuous variables, such as age and prostate volume. Comparative analysis - patients were compared based on high-risk factors and their preoperative catheterization status in order to evaluate any associations with outcomes following GLL.PVP surgery.

## Results

A total of 50 GreenLight Laser PVP surgeries were performed over a 14-month period from May 2023 to July 2024 at West Middlesex University Hospital in London. The demographic and preoperative characteristics of the patients are presented in Table 1.

**Table 1:** Patient Demographics and Preoperative Characteristics.

Values	Mean ± Standard Deviation	
Age (Years)	73.2 ± 7.80	
Prostate Volume (cc)	57.51 ± 27	
	YES	NO
Medical Management before operation	43 (86%)	07 (14%)
Long-term catheter before operation	17 (34%)	33 (66%)
High-risk factors	25 (50%)	25 (50%)

**Figure 1:**
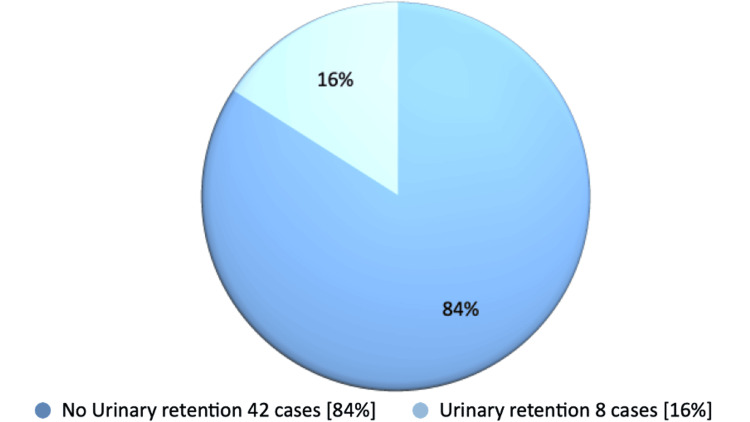
Postoperative Urinary Retention (UR) After GreenLight Laser Photoselective Vaporization of the Prostate (GLL.PVP) Surgery in patients with and without preoperative urinary retention or high-risk factors.

In our 3-6 month retrospective follow-up study of postoperative patients, we found that eight cases (16%) developed urinary retention, with an average post-void residual (PVR) volume of 446.50 mL. In contrast, 42 cases (84%) did not experience urinary retention, with an average post-void residual (PVR) volume of 105 mL and an average maximum flow rate (Qmax) of 13.5 mL/second (Figure 1). Notably, the patient without h/o urinary retention had an average preoperative PVR of 150 mL and an average Qmax of 7 mL/second. 

**Figure 2:**
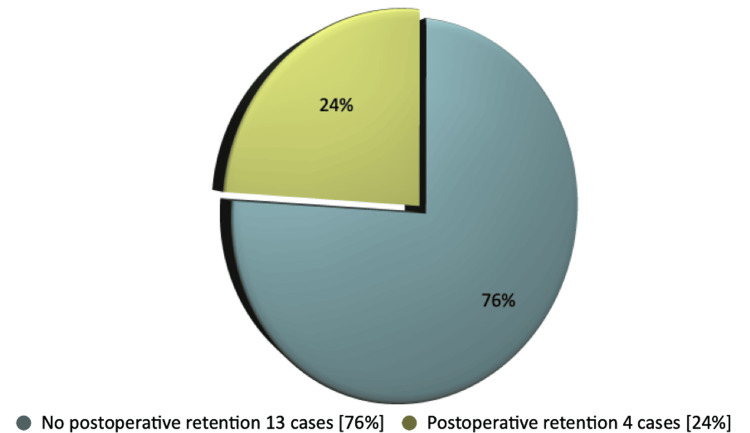
Postoperative UR after GLL.PVP operation in patients with preoperative h/o chronic urinary retention. UR: urinary retention; GLL.PVP: GreenLight Laser Photoselective Vaporization of the Prostate

Among the patients who developed urinary retention following surgery, five cases (63%) had high-risk factors beforehand, while three cases (37%) did not. In patients with preoperative urinary retention, 13 cases (76%) did not develop UR postoperatively whereas four cases (24%) developed (Figure 2). We did not find significant differences in postoperative outcomes related to varying prostate sizes during the preoperative period.

## Discussion

Our thorough study found that 84% did not develop urinary retention in 3-6 months following GLL.PVP operation, regardless of whether they had high-risk factors or used a long-term catheter preoperatively. Only 16% of total patients developed UR. Notably, in patients with a history of urinary retention before surgery, only one-fourth developed UR in our follow-up study. This result definitely revealed the efficacy of GLL.PVP operation. This result clearly demonstrated the effectiveness of the GLL.PVP operation.

The GLL.PVP treatment has proven effective in alleviating urinary symptoms related to benign prostatic hyperplasia. Strong evidence indicates that, compared to transurethral resection of the prostate, the GreenLight laser procedure is associated with shorter hospital stays, reduced need for postoperative catheterization, and better preservation of ejaculatory function after 12 months. Leading clinical experts affirm that, based on their extensive experience, the GreenLight laser procedure is a highly effective treatment option for individuals with BPH [10].

Laser therapies provide a new approach to treating BPH, with GLL.PVP gaining attention as a potential primary treatment option. This method typically uses a 532 nm green laser generated with potassium-titanyl phosphate (KTP) or lithium triborate crystals. The green laser is readily absorbed by soft tissue hemoglobin, which enhances coagulation and reduces the risk of injury to deeper tissues during vaporization. This characteristic distinguishes it from other laser modalities [11, 12, 13].

The GreenLight laser procedure is considered safe for treating prostates with a volume of up to 100 ml. Additionally, it has been determined that prostates up to 150 ml can be appropriately treated with GLL.PVP under the care of an experienced clinician [10]. The GreenLight laser procedure is a highly effective treatment for patients with large prostates and high-risk factors. It delivers rapid improvements in both subjective and objective voiding parameters, ensuring patients experience significant relief quickly [14].

The GOLIATH study is a randomized, multicenter, non-inferiority trial that compared GLL.PVP with TURP. The results indicated that both treatments were similarly effective, as measured by the International Prostate Symptom Score (IPSS), maximum urinary flow rate, and residual urine at 6 and 12 months [15, 16].

Limitations of the study

Our inclusive study offers valuable insights into evaluating postoperative urinary retention and the success rate of the GLL.PVP procedure for patients with BPH. However, we need to acknowledge some limitations. The primary limitation of our analysis is its retrospective nature, and the small sample size may hinder the generalizability of our findings. In addition, we need to assess and compare subjective voiding parameters including IPSS score in the pre and postoperative period. To gain a more comprehensive understanding, a larger, multicentric, prospective comparative study involving different modalities, including TURP, HoLEP (Holmium Laser Enucleation of the Prostate), and GLL.PVP would provide more representative data, especially considering the diverse demographics of BPH patients. Future research should prioritize conducting prospective studies with larger sample sizes and encourage collaboration across multiple centres.

## Conclusions

Our comprehensive study conclusively found that 42 cases (84%) of patients did not experience urinary retention (UR) in the follow-up after undergoing the GLL.PVP operation, irrespective of any history of urinary retention or other high-risk factors. This unequivocally demonstrates the operation's efficacy. In addition, our study findings revealed that three-quarters of patients with a preoperative history of urinary retention also did not develop UR post-surgery. The GLL PVP procedure is both safe and effective for patients with BPH, resulting in quick improvements in voiding parameters.
